# CVRriculum Program Faculty Development Workshop: Outcomes and Suggestions for Improving the Way We Guide Instructors to Embed Virtual Reality Into Course Curriculum

**DOI:** 10.7759/cureus.13692

**Published:** 2021-03-04

**Authors:** Eva Peisachovich, Lora Appel, Don Sinclair, Vladislav Luchnikov, Celina Da Silva

**Affiliations:** 1 Medical Education and Simulation, York University, Toronto, CAN; 2 Computational Arts, York University, Toronto, CAN

**Keywords:** virtual reality, faculty development, workshop, empathy, emotional intelligence, experiential education

## Abstract

Experiential education and student engagement are a main source of student attraction and retention in post secondary milieus. To remain innovative, it is imperative that universities look beyond the internet and traditional multimedia mediums and incorporate novel ways and cutting-edge technologies that can drastically change the way students and educators experience learning. The application of technology as an approach to experiential education is becoming more popular and has extensively impacted universities and other higher education organizations around the world. One approach to support this change in education delivery is to use immersive technologies such as virtual reality (VR). Our team has conducted a pilot study that focuses on embedding VR as a medium to teach empathy within higher education milieus. We began the study by conducting a pilot faculty development workshop to provide an understanding of VR and ways it can be embedded as a pedagogical approach to support curriculum design. Five faculty members from a local university were recruited to participate. Outcomes suggest that embedding VR into the curriculum is a feasible approach that provides an engaging learning environment that is effective for teaching an array of interpersonal skills. The workshop laid the foundation for future faculty training programs guiding the use of VR, prompting a dialog regarding plans for future workshops across a pan-university context.

## Introduction

As artificial intelligence, machine learning, and automation increasingly replace many of our professional tasks, human capabilities and skills, such as persuasion, social understanding, emotional intelligence, and empathy are likely to become more sought after. Unfortunately, these human-oriented “soft” skills are increasingly missing from our social context. Hence, it is important to recognize emotional intelligence as an imperative set of interpersonal skills and invest in developing and integrating mediums to both teach and embed it into curricula.

One way to develop these skills is through the use of immersive technologies, such as virtual reality (VR). Virtual reality is the term used to describe a three-dimensional (3D), computer-generated environment that can be viewed and/or interacted with by a person. At a minimum, VR systems typically utilize a head-mounted display (HMD), as the main device for a user to view and interact with a virtual world. An HMD is a display device worn on a user’s head, which includes two small displays (one for each eye) to show computer-generated imagery in 3D stereoscopy. Coupled with auditory stimuli (typically delivered through headphones that are worn by the user) and haptic feedback, VR experiences are truly immersive and elicit perceptions and behaviours similar to those one would observe in real life. Because of its ability to stimulate our senses synchronously and create the illusion of reality, VR has been termed the “empathy machine” [1].

Virtual reality as an experiential-education approach 

Experiences are what define us as humans; it is not surprising, then, that research indicates that experiential learning increases learner retention rates considerably [2]. Additionally, research on the application of 360° videos in learning suggests that immersive technology contributes to increased levels of engagement and interest in subject matter [2]. When compared with 2D visual stimuli, 360° videos result in less task-unrelated thoughts, thus benefiting a person’s cognition [3].

Given the VR’s ability to provide a visceral experience, we suspect that it can be an effective tool for experiential education, deepening the understanding of course material and introducing novel perspectives for learners [4]. VR could facilitate visual representations of complex concepts, thus increasing understanding and reducing misconceptions. As such, VR technology can be used to assist professors and guide students towards learning outcomes in higher education.

From an experiential-education perspective, VR has many benefits: it can immerse the viewer remotely into diverse environments and afford learners the ability to practice and visualize a virtual context [5]; it provides a safe and controlled learning environment; and it bridges the gap between theory and practice, and can dramatically improve upon learning by rote alone, by providing learners an opportunity to experience theoretical concepts through an interactive 3D milieu and thus engage with the context being learned or interacted with. It is also methodologically advantageous, as it makes it possible for all participants to undergo the exact same experience, since participants do not have to rely on their pre-existing schemas or biases, or use their imaginations, as they would during traditional perspective-taking tasks. Further, VR has the potential to take learning beyond the traditional online learning experience; with benefits such as enhanced engagement, improved retention, and experiential learning, this technology has the potential to revolutionize how online training programs are performed. Yet, despite its potential usefulness for higher education purposes, a systematic review of VR technology shows that it has yet to be applied as a regular act of teaching [6].

The use of VR as an educational tool is, however, making inroads in the business sector; a growing number of start-ups are creating VR training programs that will enable employees to better recognize unconscious bias [7]. Immersed in a realistic situation, the VR viewer experiences “what it feels like” to be on the receiving end of intolerance, which has been shown to draw out the empathy necessary to change negative behaviour [8]. One study found that exposing study participants to a VR experience about homelessness, rather than a paper narrative, resulted in a more enduring positive attitude towards homeless people [9].

Developing emotional intelligence to enhance academic achievement 

Emotional intelligence is defined as one’s ability to relate to another individual’s emotional experience [10]. One of the main components of emotional intelligence is expressed through the ability to manage interpersonal situations [11]. 

Therapeutic professions, such as nursing, social work, and psychology, require emotional intelligence to work in harmony with the client and their family and address patients’ emotional and physical needs [11,12]. This has resulted in a renewed focus on developing student empathy, caring, and compassion, and a concerted effort to develop students’ emotional intelligence through curriculum redesign. A study that examined whether emotional intelligence affected academic achievements found that flexibility in dealing with life situations can be a better predictor of students’ post-graduation success than grade point average [12-14].

A study of first- and second-year nursing students showed both that there is a general correlation between emotional intelligence and nursing grades and that higher emotional intelligence led to higher success of graduates in clinical work in health centres [15,16]. Emotional intelligence is also a common factor among leaders as well as innovative and effective managers [16]. Both our intuition and evidence-based research indicate that high emotional intelligence contributes to self-confidence, control, flexibility, and empathy [15,16].

CVRriculum program 

Our team developed the CVRriculum (CVR) program, in order to explore the feasibility and benefits of embedding VR into course curriculum. Our intent was to (a) report on the challenges of embedding VR into existing curriculum, (b) evaluate the benefits of introducing VR as an experiential-education medium, and, if effective, (c) create an e-library that hosts CVR-created experiences to share among students that could then deepen the learner’s understanding of empathy across disciplines and departments. The goal was to have CVR accessible remotely on the web and, for full immersiveness, viewable using VR HMD. As a first step, the team led a faculty development workshop, providing an introduction to the program and initial training to recruited instructors. 

## Technical report

**Figure 1:**
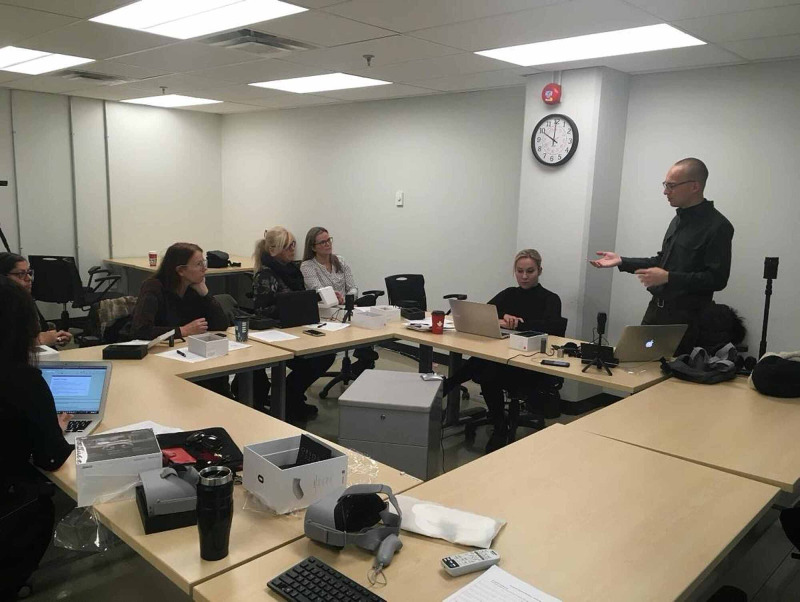
Faculty Development Workshop

A faculty development workshop was delivered in November of 2019 with the goal of introducing faculty members to VR equipment, providing relevant background on experiential education, and creating an opportunity to brainstorm how to incorporate VR into course work, as illustrated in Figure 1. 

Five faculty members from a local Ontario university were recruited through purposive sampling to participate in the workshop (four participants were teaching nursing courses and one was teaching a humanities course). These participants were expected to embed what they learned in the workshop into their courses, which cover the topics of nursing leadership, community nursing, global health, and culture and communication.

Other participants supporting the workshop included two facilitators and two research assistants (RAs). The facilitators were responsible for organizing the workshop, providing teaching materials, and explaining the purpose of the workshop and the overarching project goals. The RAs were responsible for recording the workshop (which was uploaded to the CVR website), assisting the faculty members in navigating the equipment, taking detailed observational notes about the dialogue and challenges that occurred during the workshop, and distributing and collecting consent forms and exit surveys.

Faculty were given the opportunity to engage with VR technology, share how they would embed VR into their course work, and learn about how the technology can be applied to replace a traditional assignment, such as transforming an observational essay-writing exercise into a short 360° video-documentary assignment. The objectives of the pilot faculty development workshop were to (a) provide a hands-on equipment session, during which the process of 360° video capture and VR playback was demonstrated and explained; and (b) provide examples of embedding VR into coursework through specific curriculum examples.

Each faculty member had the opportunity to experiment with the technology and received one HMD and one camera to practice with over a two-month period prior to the winter term (January 2020). Participating faculty members were also provided with the RA’s (who was supporting the implementation of the study) contact information should they need additional help experimenting with the equipment following the workshop.

VR devices 

The VR equipment for the workshop was selected for the following reasons: we found that the Oculus Go VR Headset, Yi 360° VR Camera, and Bushman Panoramic Tripod were simple to use and also offered fidelity of experience. Compared with other headsets, the Oculus Go was affordable, portable (it is both mobile and wearable and not requiring a tether), and easy to use (it requires no external hardware). Further, its built-in head-tracking module greatly improved the motion latency, thus reducing the likelihood of simulator sickness caused by the motion lag, which users often experience with other lower-end headsets like the Google Cardboard [17]. The small, lightweight Yi 360° VR camera is equipped with two 180° lenses. In addition to its ability to capture 4K 360° video, it offers the important capability of automatically splicing video together internally, without input from the user. Finally, we found the Bushman Panoramic Tripod to be ideal for 360° video capture; the tripod legs extend from the base, rather than the top, of the stand, thus minimizing the footprint captured by the 360° camera. This facilitates both 360° panoramic photography and 360° VR videography. It was our goal to minimize the technical barriers - identified during the course of the workshop as one of the main challenges - and focus on evaluating and providing suggestions for improved program implementation [18]. 

Faculty development workshop evaluation 

As this was the first time the faculty development workshop was held, the CVR team expected to identify areas of improvement. To prepare, the team planned to video-record the event (now available in https://www.yorku.ca/cvrprogram/) and take detailed observations during the workshop for future debrief and analysis. To better understand participants’ experiences, the team documented questions that arose from the faculty members, and collected feedback through an “exit survey” that included the System Usability Scale (SUS) used to evaluate comfort with the VR devices, and a nine-item questionnaire evaluating the workshop, and finally four open-ended questions regarding the effectiveness of the instruction as well as potential areas for improvement. 

The SUS was designed as a coarse but reliable tool to evaluate the effectiveness, efficiency, and satisfaction with which users can achieve specified goals in particular environments and the “ease of use” of a wide variety of technological devices and services, including hardware, software, mobile devices, websites, and applications. It consists of a 10-item questionnaire, with a five-point Likert scale of potential responses: strongly disagree, disagree, neutral, agree, and strongly agree [19,20]. We distributed the SUS questionnaire to gain an understanding of how comfortable faculty members were using the VR system (camera, headset, and tripod).

Additionally, general feedback about the workshop was garnered from a nine-item questionnaire that employed a five-point Likert scale of potential answers: poor, below average, average, good, and excellent.

Finally, four qualitative open-ended questions provided an opportunity for the faculty members to put their feedback in context and offer suggestions to improve the delivery and general approach of the faculty development workshop.

Participants were asked to think about their recent use of VR and reflect on their workshop experience when responding to the exit survey questions listed in Table 1.

**Table 1:** Faculty Development Workshop Exit Survey Using System Usability Scale

Exit Survey Statements
I think that I would like to use this system frequently.
I found the system unnecessarily complex.
I found the system was easy to use.
I think that I would need the support of a technical person to be able to use this system.
I found the various functions in this system were well integrated.
I thought there was too much inconsistency in the system.
I imagine that most people would learn to use this system very quickly.
I found the system very cumbersome to use.
I felt very confident using the system.
I needed to learn a lot of things before I could get going with the system.

Exit survey findings 

**Figure 2:**
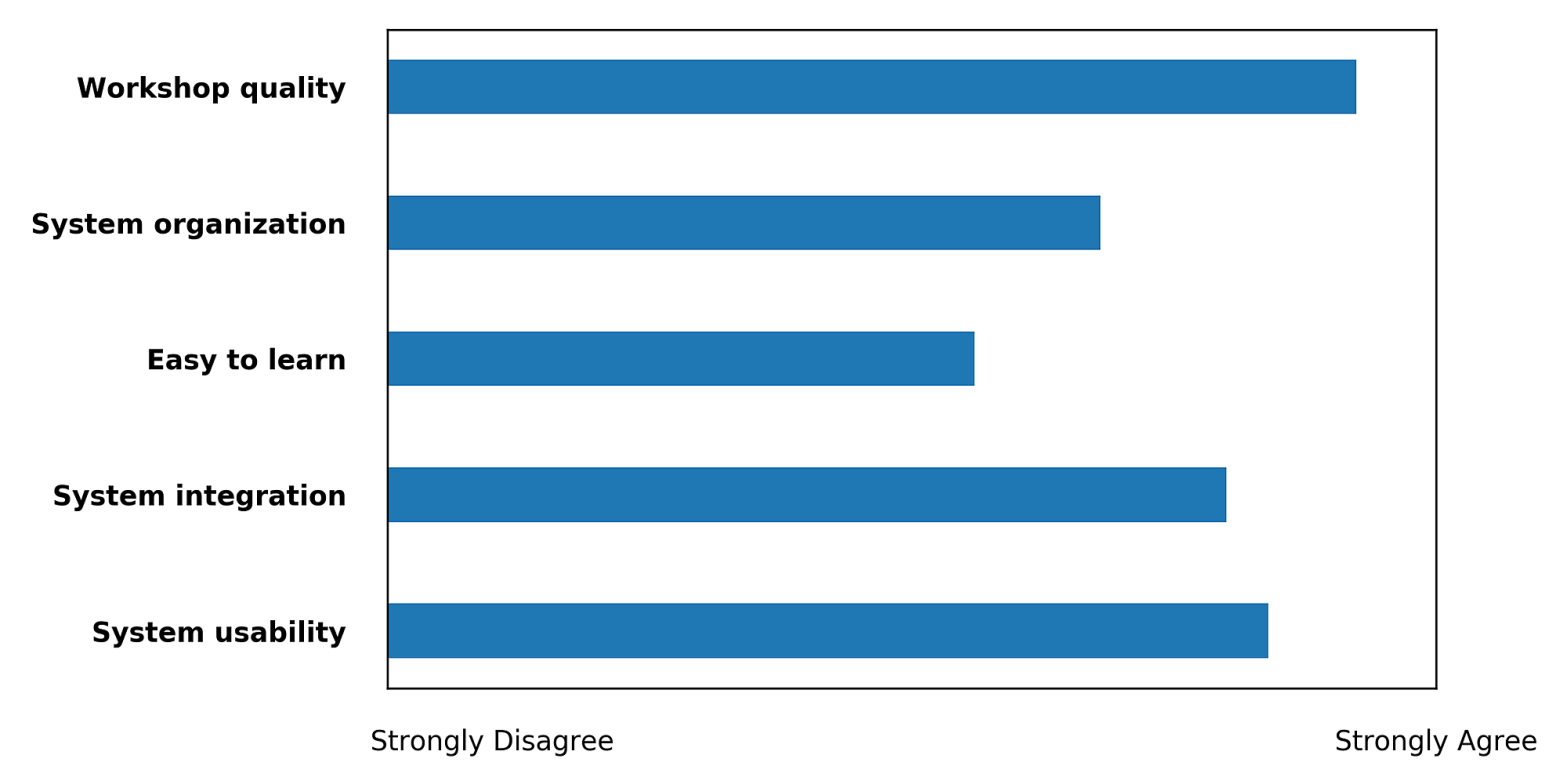
Faculty-Member Responses Regarding Virtual Reality Usability

The survey results indicate that 80% of the faculty members felt very confident using the VR system; they also agreed that the VR was easy to use and that most people would quickly learn to use the VR. All faculty members strongly agreed that they were interested in using the system frequently and that the various functions in the system were well integrated. Although none found the system too complex or cumbersome to use or reported inconsistencies in the system, two participants agreed that the support of a technical person was needed in order to be able to use this system. The responses from the faculty members regarding VR usability and workshop quality are illustrated in Figure 2. 

Additionally, general feedback about the workshop was garnered from a nine-item questionnaire that employed a five-point Likert scale of potential answers: poor, below average, average, good, and excellent. The questionnaire items are outlined in Table 2.

**Table 2:** Feedback About Workshop VR, virtual reality

Survey Statements
I found the workshop program to be . . .
I found the material provided to be . . .
I found the balance between presentations discussions and activities to be . . .
I found the time distribution of workshop to be . . .
I found the organization of the workshop to be . . .
The group work was an effective medium during the workshop.
The workshop has advanced my expertise in planning, creating, and facilitating experiential education.
I feel confident implementing VR in my teaching.
I felt the feedback received over the course of the workshop was valuable.

The faculty members reacted favourably to the workshop, responding with a good or excellent rating for all but two items: group/dyad work being an effective medium during the workshop, and confidence in implementing VR in teaching. Although all faculty members reported interest in using VR frequently, 60% rated their confidence in implementing VR in teaching as average.

Four qualitative open-ended questions at the end of the survey provided an opportunity for the faculty members to put their feedback in context and offer suggestions to improve the delivery and general approach of the faculty development workshop (see Table 3). 

**Table 3:** Additional Questions About the Effectiveness of the Instruction and Potential Areas for Improvement

Open-Ended Questions
What did you like or find MOST useful about the workshop?
What did you not like or find the LEAST useful about the workshop?
Do you have any suggestions on what to improve with the faculty development workshop or tools provided?
Do you have any additional comments?

 

Feedback regarding most useful parts of the workshop identified the following elements: (i) introduction to VR, (ii) exploration of “real-life” VR applications, (iii) overview of simulation educational theory, and (iv) opportunity to have meaningful conversations between faculty members about embedding VR into courses in place of traditional assignments.

Faculty members suggested to provide a brief VR demonstration (perhaps a video recording) prior to the workshop in order to gain some initial awareness about the equipment. The faculty members also mentioned the need to have more time to practice with the technology, specifically using the VR HMD and learning to transfer files from one device (360° camera) to the other HMD.

Another suggestion was to separate the workshop in two parts - one for addressing the technology and the other for adapting an existing assignment into a CVR deliverable.

Faculty members suggested that working as a dyad throughout the workshop would be an effective method to increase confidence and inform the implementation of VR into courses. Within working groups we found having faculty members from the same department to be more conducive to idea generation, given their common academic interests and the ability to reuse and share ideas across courses.

One participant mentioned other possibilities for VR application in the education milieu, including as a lab placement, in lieu of a clinical placement, and as a means to teach other skills, such as psychomotor skills.

Overall, the feedback was positive and suggested that the workshop was well organized and provided participants with an opportunity to envision how VR could be used in their courses in lieu of an existing assignment. 

## Discussion

The workshop explored the expanding potential VR can have on pedagogy by creating a space in which to imagine how immersive technologies can fit into curriculum.

Although the presentation delivered by the two facilitators was well received, participant feedback and debriefs from the team identified a number of ways in which we can improve the faculty development workshop in the future:

1. We plan to provide resources, such as instructional videos, ahead of the workshop in order to allow faculty members to gain an initial familiarity with the technology and its possible products. 

2. We will provide examples of past projects and the process taken to adapt traditional assignments into CVR deliverables. We intend to create separate videos as examples of how to embed VR into courses; we hope that this will address the difficulties some faculty members had in coming up with ideas about how to flip a traditional assignment into a VR assignment or how to embed VR into their teaching practices. We will ask future faculty participants if they would be willing to share their work (and assignment descriptions/rubrics and student outcomes) to help those who will participate in the future iterations. 

3. We will divide our next workshop over two days: one day to allow participants to learn further about the technology, and one day to explore project adaptation. 

4. Since participants suggested that working within a dyad would be beneficial to brainstorming and confidence, we will have faculty members work in dyads to provide comments and suggestions on each other’s work. When working in dyads, faculty can practice presenting the assignment to one another as if the other were a student in their class, and then the “learner” can report on how well they understood the assignment and deliverables. While the workshop will be delivered to a variety of disciplines, we will ensure that working dyads are composed of faculty members from the same department, as we feel their courses’ similar content will enable understanding and allow them to work on a shared VR project adaptation. 

5. we will embed a multidisciplinary brainstorming session (following the dyad work) to cross-pollinate the ideas generated within the dyads. 

Recommendations and considerations 

While the pilot CVR program identified how the use of VR can support in-class interactions and interpersonal skills among learners, we realize that given the developing social needs during a pandemic and the shift to online learning, the team should consider ways to support VR virtually. For example, the VR equipment, needs to be disinfected between uses, and its distribution introduce new processes requiring faculty coordination.

The foremost goal of the CVR program is to enhance learning; however, cost effectiveness is crucial if this educational technique is to be seen as a viable option for institutions. To be able to properly embed this approach into the university milieu, we will require a pool of equipment (HMDs, VR headsets, cameras) to supply users and to provide a technical assistance approach both to maintain and repair the hardware and to support learners with issues and questions that may arise. Our team is currently collaborating with the library department to create a “loan system” to allow users from across the university to get access (students, faculty, staff). It is also quite conceivable, given that the continuing development of VR headsets is resulting in an expanding range of options, that students will already own, or will purchase, VR headsets for personal or family use. Future studies should test delivery across the spectrum of VR devices - both low cost (often using a smartphone) and higher quality (leveraged by gaming communities) to confirm that learners’ experiences both at home and in the lab are effective and relatively consistent.

We intend to pilot the CVRriculum within various disciplines and contexts, where these virtual experiences can replace clinical-placement opportunities. In addition to health care (where VR experiences can be used to explore patient-centred care and gain understanding of the patient condition), this technology can be applied to education, social work, psychology, and the humanities, where simulations can support learners hone communication and interpersonal skills.

## Conclusions

Overall, the faculty development workshop was successful; it laid a foundation for future training programs of this nature and allowed our team to further refine the workshop to meet the needs of faculty members wanting to embed VR technology in their curricula. The workshop participants were satisfied, as evidenced by the feedback from the exit survey confirming that the faculty participants were satisfied and gained the knowledge and confidence to embed VR into their courses. This experience has provided the team with a framework or “blueprint” for a cross-disciplinary program to provide faculty members with training using VR, which will afford students an opportunity to practice and develop competencies such as emotional intelligence and empathy in their respective fields.

Immersive instructional technologies using VR applications can provide benefits commonly associated with experiential education, such as the development of empathy. Hence, our goal is to further explore its impact on the teaching and learning process. We hypothesize that the immersion of faculty development workshops associated with the CVR program may contribute to sustained growth in online learning and that embedding VR will provide opportunities both for fostering student success and for enabling curriculum synergies.
